# A Spike Train Distance Robust to Firing Rate Changes Based on the Earth Mover’s Distance

**DOI:** 10.3389/fncom.2019.00082

**Published:** 2019-12-10

**Authors:** Duho Sihn, Sung-Phil Kim

**Affiliations:** Department of Human Factors Engineering, Ulsan National Institute of Science and Technology (UNIST), Ulsan, South Korea

**Keywords:** neural spike train, spike train distance, Earth Mover’s Distance, temporal coding, synchrony

## Abstract

Neural spike train analysis methods are mainly used for understanding the temporal aspects of neural information processing. One approach is to measure the dissimilarity between the spike trains of a pair of neurons, often referred to as the spike train distance. The spike train distance has been often used to classify neuronal units with similar temporal patterns. Several methods to compute spike train distance have been developed so far. Intuitively, a desirable distance should be the shortest length between two objects. The Earth Mover’s Distance (EMD) can compute spike train distance by measuring the shortest length between two spike trains *via* shifting a fraction of spikes from one spike train to another. The EMD could accurately measure spike timing differences, temporal similarity, and spikes time synchrony. It is also robust to firing rate changes. Victor and Purpura (1996) distance measures the minimum cost between two spike trains. Although it also measures the shortest path between spike trains, its output can vary with the time-scale parameter. In contrast, the EMD measures distance in a unique way by calculating the genuine shortest length between spike trains. The EMD also outperforms other existing spike train distance methods in measuring various aspects of the temporal characteristics of spike trains and in robustness to firing rate changes. The EMD can effectively measure the shortest length between spike trains without being considerably affected by the overall firing rate difference between them. Hence, it is suitable for pure temporal coding exclusively, which is a predominant premise underlying the present study.

## Introduction

A spike train is the sequence of neuronal firing timings, where a spike refers to the firing of an action potential. The temporal pattern of a spike train encodes information in various ways. Besides firing rates, the temporal pattern of spike timings also carries important information about brain functions. For instance, it has been shown that temporal patterns encode the information of auditory (Machens et al., 2001; Narayan et al., 2006; Wang et al., 2007; Fukushima et al., 2015; Krause et al., 2017), gustatory (Di Lorenzo and Victor, 2003), motor (Vargas-Irwin et al., 2015), olfactory (MacLeod et al., 1998), somatosensory (Harvey et al., 2013), vestibular (Jamali et al., 2016), and visual (Mechler et al., 1998; Victor and Purpura, 1998; Reich et al., 2001; Carrillo-Reid et al., 2015) systems, as well as behavioral adaptation (Logiaco et al., 2015) and sleep (Tabuchi et al., 2018).

One of the many approaches for studying a temporal firing pattern is to measure the dissimilarity between a pair of spike trains, which is often represented by the spike train distance. The measurement of spike train distances can be designed to represent rate coding, temporal coding, or both. Several methods to measure a spike train distance have been proposed. Victor and Purpura introduced a cost-based distance that assigns a cost to shifting, adding, or deleting a spike (Victor and Purpura, 1996). In this method, the spike train distance is defined as the minimum of all possible sums of costs. The Victor and Purpura distance depends on a time-scale parameter where the smaller value of the time-scale parameter emphasizes temporal coding while the larger value does rate coding. van Rossum (2001) also developed a spike train distance that measures a difference between spike trains convolved with exponential functions. Most spike train distances are rate-sensitive, reflecting an overall rate difference between spike trains to a certain extent even with an extreme time-scale parameter (Satuvuori and Kreuz, 2018). Here, the overall rate denotes the total number of spikes in a spike train divided by the time length of the train. If one aims to measure a distance between a pair of spike trains independent of the overall rate difference, which we call as purely timing-sensitive, the distance should reflect only a difference of spike timing distributions, no matter how different the overall firing rate is between trains.

A purely timing-sensitive spike train distance is important to neuroscience studies on temporal coding, which assumes that neurons code information in spike timing patterns (Tabuchi et al., 2018). If a spike train distance is rate-sensitive, it would be difficult to clarify whether a given result from a neural spike train analysis is based only on the temporal information. It has been suggested that precise spike timing plays a crucial role in neural information processing (Butts et al., 2007; Gollisch and Meister, 2008; Johansson and Flanagan, 2009).

Kreuz et al. (2007) developed the rate-sensitive ISI-distance, a spike train distance based on a ratio between the inter-spike intervals of two spike trains. This was followed by the SPIKE-distance, a complementary distance which is still sensitive to rates but with a heightened sensitivity to spike timing (Kreuz et al., 2013). Finally, by removing rate dependence from the SPIKE-distance, Satuvuori et al. (2017) proposed the RI-SPIKE-distance as a distance purely sensitive to timing. The spike train distances developed so far have been used in a number of studies for the analysis of neural firing patterns (MacLeod et al., 1998; Mechler et al., 1998; Victor and Purpura, 1998; Machens et al., 2001; Reich et al., 2001; Di Lorenzo and Victor, 2003; Narayan et al., 2006; Wang et al., 2007; Harvey et al., 2013; Fukushima et al., 2015; Logiaco et al., 2015; Vargas-Irwin et al., 2015; Jamali et al., 2016; Krause et al., 2017).

Nevertheless, in an intuitive manner, one of the desirable properties of *distance* would be a capability to measure the shortest length between two objects. In this sense, the previous methods to measure spike train distance have not clearly represented the shortest length because they do not minimize the distance value explicitly, except for the Victor and Purpura distance, which explicitly measures the shortest length (Victor and Purpura, 1996). Yet, although this distance represents the minimum cost related to the shortest length, it suffers from the fact that distance output is not unique because this approach employs a parameter (i.e., *q* in their model) assigned to the cost for spike time shift. Thus, distance output depends on how *q* is determined. This property can be advantageous for some spike train analyses, but not in other cases that need a unique value (Chicharro et al., 2011). In the present study, therefore, we adopt the Earth Mover’s Distance (EMD) to measure spike train distance with a unique shortest length.

The EMD is also called the Wasserstein metric, which defines the distance between a pair of probability distributions. Here, a metric refers to a distance satisfying non-negativity, symmetry and the triangle inequality. It measures the minimal cost based on an underlying distance taken to transfer from a probability distribution to another. It initially dealt with transportation problems (Kantorovich, 1940) and later modified toward today’s form (Vaserstein, 1969). The EMD also has been implemented as an algorithm in the field of computer science for the comparison between two images (Rubner et al., 2000). The main idea underlying the EMD is that the shortest distance between two objects is equal to the length of the shortest delivery path from one object to the other. For neural spike data, *delivery* in a spike train operates by moving a part of the spike train from one location to another, with a goal to match one spike train with the other. A delivery path length is then calculated by summing the delivery distance between two locations multiplied by the amount of a delivered part. If we deal with a spike train as a distribution with a sum of 1, then the EMD measures a unique shortest distance between a pair of spike trains in a non-parametric way. A notable difference of the EMD from that of Victor and Purpura (1996) is that delivery in the method of Victor and Purpura moves an entire spike at once while delivery in the EMD can move a part of a spike.

Spike train distance can be used for both rate coding and temporal coding (Satuvuori and Kreuz, 2018). Rate coding accounts for the firing rate profile of neuronal spike trains while temporal coding relates to temporal patterns. In our development of spike train distance, we focus on a particular aspect of the firing rate profile, an overall firing rate difference between spike trains, whereas we refer a temporal pattern of spike train to the distribution of spike timings in time within a spike train. Specifically, the temporal pattern focuses on the pattern of a spike timing distribution as a function of time, not on how many spikes occur in any particular time window. For example, a spike train with spike timings at (0, 1, 10) has a similar temporal pattern to another spike train (0, 0.1, 0.9, 1, 10, 10.1) while their overall firing rates are different. In the case of temporal coding, spike train distance is often used to measure the dissimilarity of temporal patterns of neuronal spike trains, which may not be explained by rate coding alone. For such a case, measurements of spike train distance need to be independent of firing rate changes. On the other hand, if a spike train distance is sensitive to firing rate changes, it may be ambiguous whether the analysis results reflect changes of mere temporal patterns or a mixed effect of firing rates. Hence, robustness against firing rate changes should be a desired property of spike train distances in pure temporal coding studies. Previous methods for spike train distance have not focused much on this robustness except the one by RI-SPIKE-distance (Satuvuori et al., 2017). As such, the EMD is chosen here to ensure robustness to firing rate changes. The EMD can measure spike distance robust to the overall firing rate difference between spike trains because the EMD normalizes the total amount of spikes in a spike train to 1, making the overall firing rate of each spike train equal.

In this study, we employ the EMD as a spike train distance for pure temporal coding research. Then, we compare the EMD with several other spike train distances using neural spike data generated from a set of simulations. The simulations are designed to evaluate the performance of the spike train distances with respect to essential aspects of temporal patterns, including spike timing differences, temporal similarity, and spike time synchrony, as well as the robustness against firing rate changes in spike trains to deal with pure temporal coding. In this study, we refer temporal coding to a scheme to represent a spiking probability as a function of time. It is different from a time-varying firing rate as it does not reflect actual firing rates over time. In several simulation tests, we evaluate how various spike train distance methods, including the proposed one, represent pure temporal coding using a spike generation probabilistic model, in which a spiking probability varies with time independent of the number of spikes. This includes the test of the robustness of each method against firing rate changes by alternating the total number of spikes while maintaining temporal coding unchanged. The advantages of the EMD in pure temporal coding research are demonstrated by the simulation results. However, it should be noted that these advantages are not directly transferable to rate coding.

## Materials and Methods

### The Earth Mover’s Distance as a Spike Train Distance

Two different spike trains may contain a different number of spikes. However, the total number of spikes of each spike train should be equalized to measure the distance between them based only on shifting spikes in time. Victor and Purpura (1996) solved this problem by assigning a cost to adding/deleting a spike and to shifting a spike in time. However, this solution cannot produce a unique distance because it varies with the ratio of two different costs. To address this shortcoming, in the proposed method, we first define a spike train in which each spike is assigned a fixed quantity of 1. Then, we normalized individual spikes by the total number of spikes, N, so that each spike’s quantity becomes 1/N after normalization. For the normalization, we consider a spike train as a function *f* of time *t* such that

(1) $$f\,\left(x\right)=\left\{ \begin{array}{c} \frac{1}{N},\mathrm{if}\,a\,\mathrm{spike}\,\mathrm{occurs}\,\mathrm{at}\,\mathrm{time}\,t\\ 0,\text{otherwise} \end{array}\right.$$

Where *N* is the number of spikes in the spike train. The overall summation of *f* must be one except the case of *N* = 0. Hereafter, a spike train will be expressed as functions *f* or *g*.

In our method, the EMD between *f* and *g* proposed in Rubner et al. was adjusted for one-dimensional data (i.e., a spike train) with a constraint that the sum of *f* or *g* should be equal to 1 (Rubner et al., 2000). The EMD is described as follows. We first rewrite the spike trains, *f* = {(*x*_1_, 1/*N*), (*x*_2_, 1/*N*),…,(*x*_N_,  1/*N*)} and *g* = {(*y*_1_, 1/*M*),(*y*_2_, 1/*M*),…,(*y*_*M*_, 1/*M*)} from Eq. 1 where *x*_i_ and *y*_j_ are a sequence of spike timings. Let *d*(*x*_i_,*y*_j_) be an absolute difference between two spike timings *x*_i_ and *y*_j_. Let *ξ*_ij_ be a flow (amount of delivery) from *x*_i_ to *y*_j_ and let Ξ = [*ξ*_ij_] be a matrix of these flows (amount of deliveries) such that it transports *f* to *g* satisfying the following conditions: (1) *ξ*_ij_ is non-negative; (2) $\sum_{i=1}^{N}\xi_{\text{ij}}\leq 1/M$, $\sum_{j=1}^{\text{M}}\xi_{\text{ij}}\leq 1/N$; and (3) $\sum_{i=1}^{N}\sum_{j=1}^{\text{M}}\xi_{\text{ij}}=1$. Condition 1 fixes the direction of the delivery from *i* to *j*. Condition 2 indicates an effective delivery in the sense that it does not take back what has been delivered. Condition 3 indicates that it delivers the entire spike train. The transportation here means that it makes *f* equal to *g* by moving parts of *f*. Then, the EMD between *f* and *g* is given by

$$\text{EMD}\left(f,g\right)=\min\{\sum_{i=1}^{N}\sum_{j=1}^{M}d\left(x_{i},y_{j}\right)\xi_{i\,j}:\Xi$$

(2) $$=\left[\xi_{\mathrm{ij}}\right]\mathrm{satisfies}\mathrm{conditions}\mathrm{above}\}$$

**FIGURE 1:**
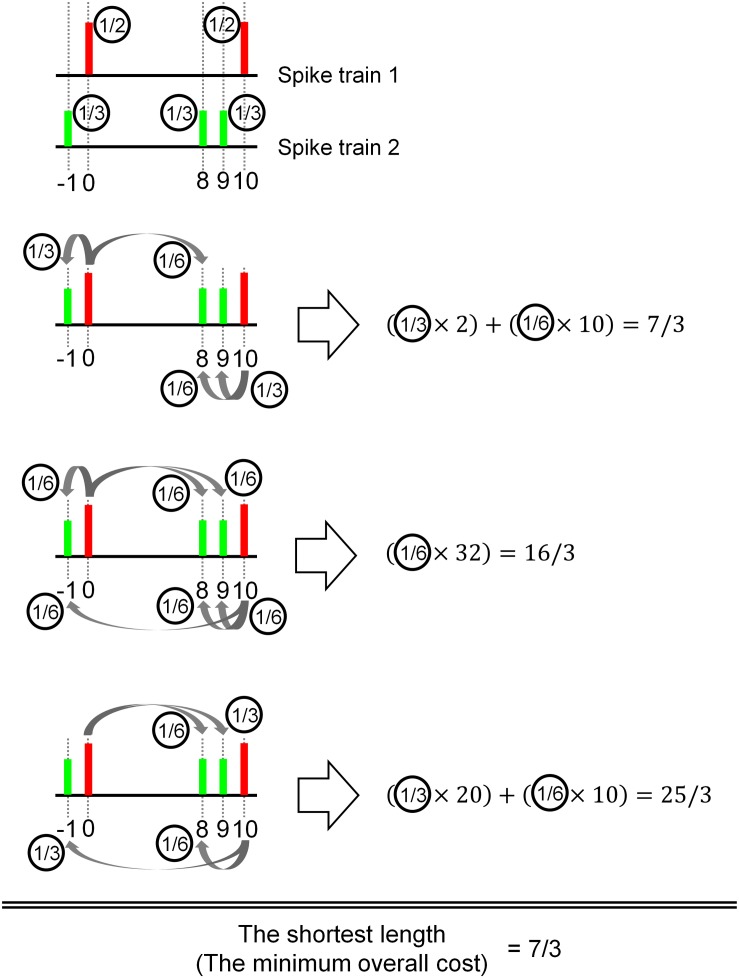
Illustration of the basic concept of the Earth Mover’s Distance (EMD) to measure a distance between spike trains. The objective is to take the smallest value among all possible delivery (flow, terminology in EMD) path lengths between two objects (red and green). In this illustrative example, the red object (e.g., spikes) is delivered to the green object in three possible paths. Assuming the size of the red object is 1, the path length is calculated by delivery distance (in time) multiplied by the amount of delivery (i.e., size of the object). It is also possible to deliver only a fraction of the object, as shown in the third case. If there are multiple deliveries toward the target object, the final delivery path length is the sum of individual delivery path lengths.

This concept of spike train distance is illustrated in Figure 1.

When *N=0* (i.e., no spike in the train), the proposed method cannot calculate the distance directly. However, it can deal with such a case indirectly if we consider a spike train with no spike similar to a spike train with spikes everywhere so that the distance of it to any other trains becomes irrelevant to a certain spike timing pattern. Let *f*_*0*_ be a spike train with no spike and let *g* be another spike train to be compared. To calculate *d*(*f*_0_,*g*), let *f*_n_ be a spike train with *n* spikes generated from a uniform probability distribution defined on a certain bounded analysis domain. The bounded analysis domain prevents the distance from increasing to infinity, although the distance measurement depends on how the analysis domain is determined. Then, the EMD calculates $d\,\left(f_{0},g\right)=\lim_{n\to \infty }\text{E}\,\left(d\,\left(f_{\text{n}},g\right)\right)$ where E(⋅) indicates an expected value. To deal with an empty spike train in the EMD, we attended to an idea that there was also no information about spike timing if spikes are everywhere, uniformly distributed. It means that a spike train with one spike at a specific location holds more information about spike timing than a spike train with uniformly distributed spikes. In this regard, an empty spike train would be more similar to a spike train with uniformly distributed spikes at every location than a spike train with one spike.

The EMD is a mathematical metric, that is, it satisfies the three conditions: non-negativity, symmetry and the triangle inequality (Rubner et al., 2000). This property shows that the EMD conforms to our intuition about distance. Moreover, from the fact that the EMD is calculated solely based on spike timing data, it can be seen that the EMD is the shortest length based on spike timing between two spike trains. The EMD is calculated in a non-parametric way so that it produces a unique value. Due to its non-parametric approach, the EMD can avoid the dependency of distance outcomes on parameters.

Moreover, there is an efficient way to calculate the restricted version of the EMD as follows (Cohen, 1999). Let *F* and *G* be the cumulative functions of *f* and *g*, respectively. Then, the EMD is given by

(3) $$\text{EMD}\,\left(f,g\right)=\int_{-\infty }^{\infty }\left|F\,\left(t\right)-G\,\left(t\right)\right|\,\text{d}\,t$$

**FIGURE 2:**
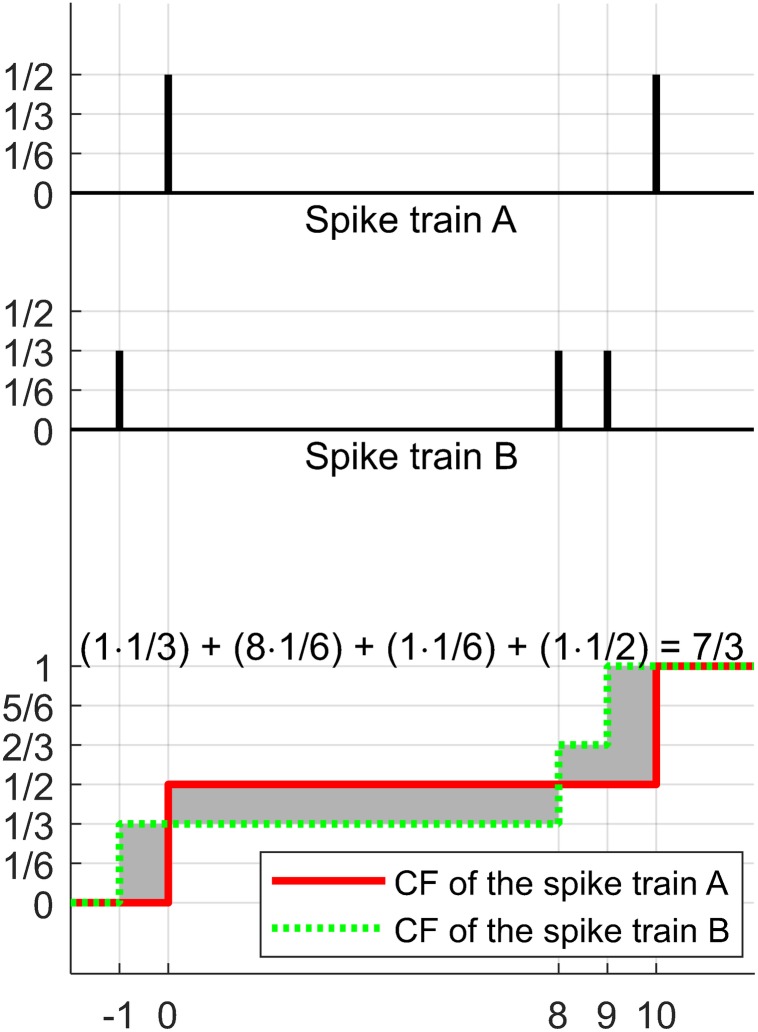
Illustration of the calculation procedure of the EMD described in Cohen (1999). The distance between two spike trains, A and B, is calculated. Initially, the non-negative values are assigned to every spike such that the sum of the values in each train is equal to 1 (e.g., 1/2 for each spike in A or 1/3 for each in B). The next step is to produce the cumulative functions (CF) for each spike train (red bold line indicates the CF of spike train A and green dotted line indicates the CF of spike train B). The next step is to integrate the absolute difference between the two CFs (gray shading area). The final result of the calculation procedure is 7/3.

An example of the calculation procedure above is illustrated in Figure 2.

### Relationship With Other Measures

The Kullback–Leibler divergence is a distance between two probability distribution functions. Therefore, the Kullback–Leibler divergence computes the difference between two functions at every point on the domain of a random variable, similar to the EMD computation as shown in Eq. 3. However, a difference between the Kullback–Leibler divergence and the EMD is that the EMD depends not only on the difference between the functional values but also on the distance between the points on the domain while the Kullback–Leibler divergence does not. In Eq. 3, the EMD is calculated by (the difference between functions) × (the length to which the difference is maintained), so that the EMD is based on spike timing difference unlike the Kullback–Leibler divergence.

In the Victor-Purpura distance, spikes are shifted if the distance between spikes is small or added/deleted if the distance is large, depending on the time-scale parameter q. On the other hand, in the EMD, no matter how large the distance is, the spikes are always shifted. This indicates that the EMD can be viewed as similar to the Victor-Purpura distance with an extremely high cost of adding/deleting spikes. But, since the parameter q of the Victor-Purpura distance controls the time shift cost only, not adding/deleting explicitly, imposing a high cost on adding/deleting spikes can be implemented by selecting a very small value for q. Consequently, the Victor-Purpura distance with a small time-scale parameter (q) becomes similar to the EMD, with an emphasis on temporal coding.

If two spike trains have the same number of spikes, *N*, and the Victor-Purpura distance does not take the option of the cost for adding/deleting spikes, the Victor-Purpura distance and the EMD are exactly the same with the time-scale parameter q = 1/N. Hence under those conditions, the EMD can be considered as the average displacement of the spikes.

When two spike trains have different numbers of spikes, the EMD still calculates the average displacement of the spikes to some extent: the displacement of the part of a spike instead of an entire spike. The displacement of the part of a spike only reflects the temporal difference between spike trains. In contrast, the Victor-Purpura distance works in a different way due to the option of the cost for adding/deleting spikes. Since the cost for adding/deleting spikes directly correlates with a difference in the number of spikes between trains, the Victor-Purpura distance can reflect the rate difference. Hence, it has been pointed out that the Victor-Purpura distance is suitable for rate coding but not for temporal coding if the number of spikes is quite different between spike trains (Satuvuori and Kreuz, 2018).

When two spike trains, *f* and *g*, have the same number of spikes, we can describe the Victor-Purpura distance without the option of adding/deleting spikes with respect to the EMD as follows: $d_{\text{EMD}}\,\left(f,g\right)=\lim_{q\to 0}\left(1/q\right)\,d_{\text{VP}\,\left[q\right]}\,\left(f,g\right)$, where *d*_*EMD*_ indicates the EMD between *f* and *g* and *d*_*VP[q]*_ indicates the Victor-Purpura distance with the time-scale parameterq. Even if f and g have a different number of spikes, the description above holds if the Victor-Purpura distance is applied to the normalized spike train as in the EMD.

### Evaluation

Our new spike train distance was compared to four existing spike train distances: (1) the Victor-Purpura distance (Victor and Purpura, 1996) with parameter values, q = 0.1, 0.2, …, 12.8 s^–1^; (2) the van Rossum distance (van Rossum, 2001) with parameter values, τ = 1, 2, …, 16 s. Note that an alternative calculation method (Houghton and Kreuz, 2012) was used here instead of the original one (van Rossum, 2001); (3) the SPIKE-distance (Kreuz et al., 2013); and 4) the RI-SPIKE-distance (Satuvuori et al., 2017).

The tested time-scale parameters of the Victor-Purpura distance and the van Rossum distance were determined as follows. For the Victor-Purpura distance parameter q, the time range of a spike train in which we performed the analysis was set to 0 - 10 s. Then we opted for values of q varying between two opposite cases: q = 0.1 s^–1^ and q = 12.8 s^–1^. The smallest q = 0.1 s^–1^ in the Victor-Purpura distance made the metric focus on a “spike timing shift” by assigning a cost of 1 to add/delete each spike, whereas it costed at most (q = 0.1 s−1)×(10s) = 1 for time-shifting a spike. Then, the value of q was increased by a factor of two up to the largest q = 12.8 s^–1^, which turned the algorithm to focus on “spike adding/deleting” by increasing the cost for time-shifting such as (q = 12.8 s−1)×(1s) = 12.8 even for shifting a spike by 1 s.

Similarly, for the van Rossum distance, the smallest value of … = 1 s makes the convolved range narrow by setting the width of the exponential function to 1 s. Then, the value of … was increased by a factor of two up to the largest value of … = 16 s, which makes the convolved range cover the overall spike train by setting the width of the exponential function to 16 s.

Taking spike counts into dissimilarity is a key difference between the EMD and the Victor-Purpura distance or the van Rossum distance. In fact, while the EMD is focused on temporal coding, both the Victor-Purpura distance and the van Rossum distance cover from a mixture of temporal coding and rate coding to pure rate coding by varying the time scale parameter q or …, as they are so designed originally. We demonstrated such differences between the EMD and the Victor-Purpura distance or the van Rossum distance in the simulations (see section “Results”).

A comparison of the five spike train distances was conducted to assess how well each distance represented three aspects of similarity between spike trains: spike timing difference, temporal similarity, and spike time synchrony. Furthermore, each distance’s robustness to changes in firing rates was examined for temporal similarity and spike time synchrony.

To avoid potential errors while replicating the existing distance calculation procedures, we directly utilized the available source code for each distance. The code to calculate the Victor-Purpura distance was obtained from http://www-users.med.cornell.edu/~jdvicto/spkdm.html. The code for the van Rossum distance was from http://wwwold.fi.isc.cnr.it/users/thomas.kreuz/images/vanRossum.m. The codes for both the SPIKE-distance and the RI-SPIKE-distance were from http://wwwold.fi.isc.cnr.it/users/thomas.kreuz/Source-Code/cSPIKE.html.

For the calculation of the SPIKE-distance and the RI-SPIKE-distance, we always set the time range of the underlying dissimilarity profiles exactly equal to the spike generation interval.

#### Spike Timing Difference

**FIGURE 3:**
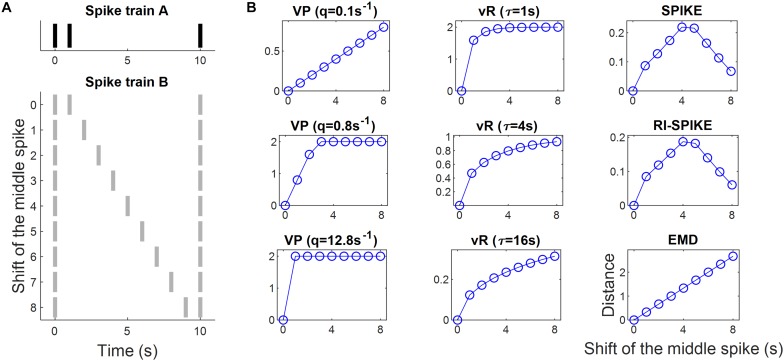
Spike train distance results for the measurement of spike timing differences. **(A)** Spike train A is fixed whereas spike train B is changed as the location of a middle spike is shifted from left to right so that the spike timing difference between A and B increases linearly. **(B)** The spike train distance results of each of the five distances: the Victor-Purpura distance, the van Rossum distance, the SPIKE-distance, the RI-SPIKE-distance, and the EMD. The horizontal axis represents the amount of the shift of the middle spike in train B. The Victor-Purpura distance showed a linear increase in distance only for a certain parameter (e.g., *q* = 0.1 s). The van Rossum distance showed an increase in distance non-linearly but monotonically. The SPIKE-distance and the RI-SPIKE-distance did not show monotone increases. The EMD showed a linear increase as the spike timing difference increased. VP, Victor-Purpura distance; vR, van Rossum distance.

A pair of spike trains with three spikes each was synthesized to test spike timing difference. The locations of the first and third spikes were fixed and matched between the trains. The second spike of the first train was fixed close to the first spike. Then, the location of the second spike of the second train was moved toward the third spike. This test paradigm was performed in the previous study by Kreuz et al. (2011) to compare several distances. We adopted it here with the inclusion of the van Rossum distance, the RI-SPIKE-distance, and the EMD. In the test, we located the first spike at 0 s and the third at 10 s in the two trains. The second spike of the first train was fixed at 1 s. Then, the second spike of the second train was moved from 1 s to 9 s in steps of 1 s (see Figure 3A). We measured the distance for each shift of the second spike of the second train.

#### Temporal Similarity and Robustness to Firing Rate Change

**FIGURE 4:**
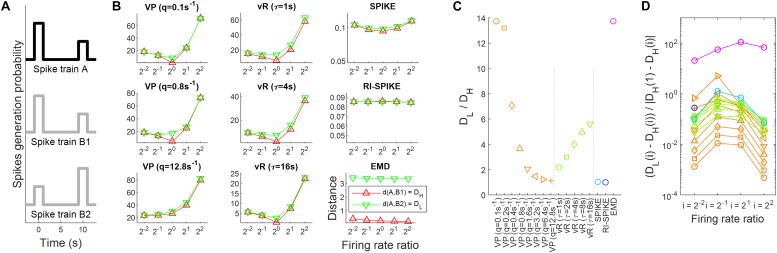
Spike train distance results for the measurement of temporal similarity. **(A)** The probabilistic models of spike generation for spike trains A, B1, and B2 are described. In the simulation, spike trains A and B1 share the same probabilistic model whereas spike trains A and B2 have different probabilistic models. Accordingly, the temporal similarity is high between A and B1, but low between A and B2. **(B)** The spike train distance results of each of the five distances as the ratio of firing rates between the spike trains varies from 2^– 2^ to 2^2^. The red lines represent distances between the spike trains A and B1 and green lines represent those between A and B2. It is clearly shown that the variability of distances by changes in the ratio is larger than that by changes in the temporal similarity for the four distances, including the Victor-Purpura, the van Rossum, the SPIKE- and the RI-SPIKE- distances. In contrast, the distances calculated by the EMD remain almost unchanged as the ratio changes, being robust to the firing rate change. **(C)** Results of spike train distance for measuring temporal similarity. *D*_L_ is a distance with a low temporal similarity, and *D*_H_ is a distance with a high temporal similarity. **(D)** Quantification of robustness as the firing rate changes. D_L_(i) is the distance with a low temporal similarity when the firing rate ratio is *i*, and D_H_(i) is the distance with a high temporal similarity when the firing rate ratio is *i*. The results of the RI-SPIKE-distance partly disappear because of negative values. VP, Victor-Purpura distance; vR, van Rossum distance.

A simulation experiment was performed to test the robustness of each distance against firing rate changes when measuring temporal similarity between spike trains. Spike trains were generated according to a simple probabilistic model. The probabilistic model was built following a certain firing rate profile. Temporal similarity would increase if a pair of spike trains were generated from a probabilistic model sharing a similar profile and decrease if the profiles become more dissimilar. Note that temporal similarity describe here depends only upon firing rate profiles, not firing rates themselves. The probabilistic model used here consisted of two intervals where each interval had a non-zero probability of containing a spike. Spikes in the intervals were randomly generated from a uniform distribution centered at 0 s and 10 s with a halfwidth of 1 s. Then, we built three spike trains denoted as spike trains A, B1, and B2. In the probabilistic model of spike train A, the probability of generating a spike in the first interval was twice as high as that in the second interval. Spike train B1 had the same probabilistic model as spike train A. On the other hand, it was reversed in spike train B2 such that the probability of generating a spike in the second interval was twice that in the first interval (see Figure 4A). Hence, the distance between A and B1 should be smaller than that between A and B2, because temporal patterns would be more similar between A and B1 than between A and B2.

To test the robustness of the distances against firing rate changes, we varied the number of spikes in the trains. We first set the number of spikes in A to 2^3^ × 3, where 2^3^ spikes were generated three times (twice in the first interval and once in the second interval). Then, five levels of the number of spikes were used to vary the firing rates in B1 or B2. The number of spikes in B1 or B2 was varied as 2^1^×3, 2^2^×3, 2^3^×3, 2^4^×3, and 2^5^×3, making the spike count ratios of A to B1 or B2 2^−2^, 2^−1^, 2^0^, 2^1^, and 2^2^. If a spike train distance is robust to firing rate changes, distance variability over all the ratios should be negligible compared to the difference in distance between A to B1 and between A and B2. We calculated the difference in distances between these two pairs (A and B1, A and B2) using each of the five distances by varying the firing rates in B1 or B2.

#### Spike Time Synchrony and Robustness to Firing Rate Change

**FIGURE 5:**
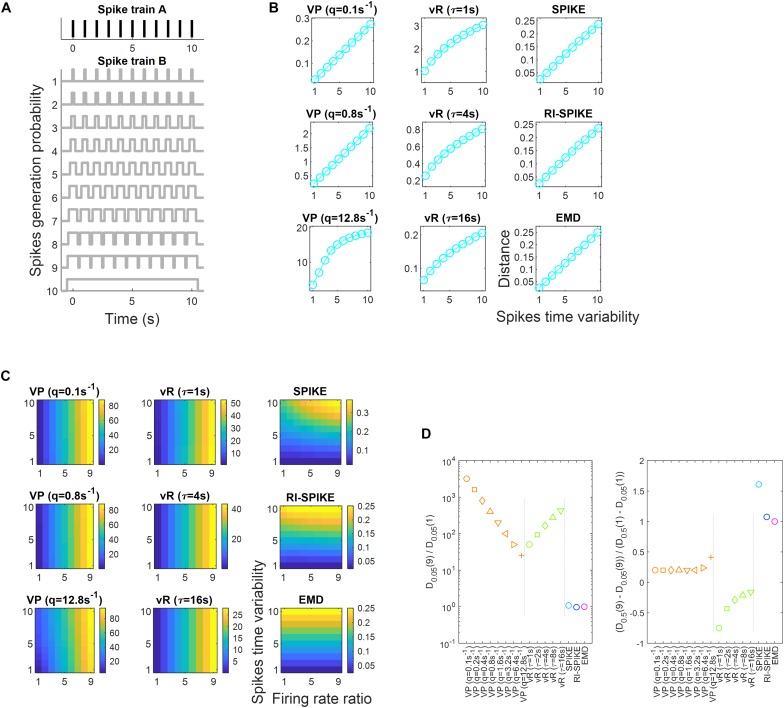
Spike train distance results for the measurement of spike time synchrony. **(A)** Spike train A is fixed to have equally spaced eleven spikes. Spike train B is generated by a probabilistic model of spike generation with various spike timing jitter. The spike timing jitter is manipulated by increasing the halfwidth of eleven uniform distributions each centered at the spike timing of train A. Spike time synchrony between A and B decreases as spike timing jitter increases. **(B)** The spike train distance results of the five distances as the ratio of firing rates of B over A are equal to 1. All the distances exhibit approximately linear increases with increases in spike timing jitter. **(C)** The spike train distance results of each of the five distances as ratios of firing rates of B over A increase from 1 to 9. The index of the vertical axis corresponds to the index of the spike trains in panel **(A)**, where increasing index number indicates increasing spike timing jitter. Distances proposed by Victor-Purpura and van Rossum are significantly affected by the variation in the firing rate ratio, whereas those proposed by the SPIKE-distance, the RI-SPIKE-distance and the EMD are not. **(D)** Results of spike train distance for measuring spike time synchrony. *D*_k_(*n*) is the distance when the firing rate ratio of one spike train to another was *n*, and *k* denotes the halfwidth of the uniform distribution in the spike train. VP, Victor-Purpura distance; vR, van Rossum distance.

Another simulation experiment was performed to test the robustness of each distance against firing rate changes when measuring spike time synchrony between spike trains. To this end, a pair of spike trains, denoted as A and B were synthesized. Spike train A was generated to contain eleven equally spaced spikes discharged at 0 s, 1 s, …, 10 s. Spike train B was generated according to a probabilistic model, consisting of eleven uniform distributions centered at 0 s, 1 s, …, 10 s. Then, we varied the halfwidth of these uniform distributions across ten levels to manipulate the degrees of spike timing jitter; the halfwidth was set as 0.05 s, 0.1 s, …, or 0.5 s (see Figure 5A). As the halfwidth was increased, spike timing jitter increased, which was likely to desynchronize spike timing more between A and B. It would then result in an increase in the distance between A and B.

Similar to section “Temporal Similarity and Robustness to Firing Rate Change,” we varied the number of spikes in B to test the robustness of the distance to firing rate change. The number of spikes in B varied across nine levels to reflect firing rate changes. It varied as 1 × 11, 2 × 11, …, and 9 × 11 (the first number in the product indicates the number of spikes randomly generated in each interval of B) so that the ratios of A to B became 1, 2, …, and 9, respectively. We expected that if the spike train distance was robust to firing rate changes, variability in the distance across the ratios should be negligible compared to variability in distance according to different degrees of spike timing jitter. We calculated the distances between A and B for each degree of spike timing jitter for each firing rate level in B.

#### Comparison With Victor and Purpura’s Distance

**FIGURE 6:**
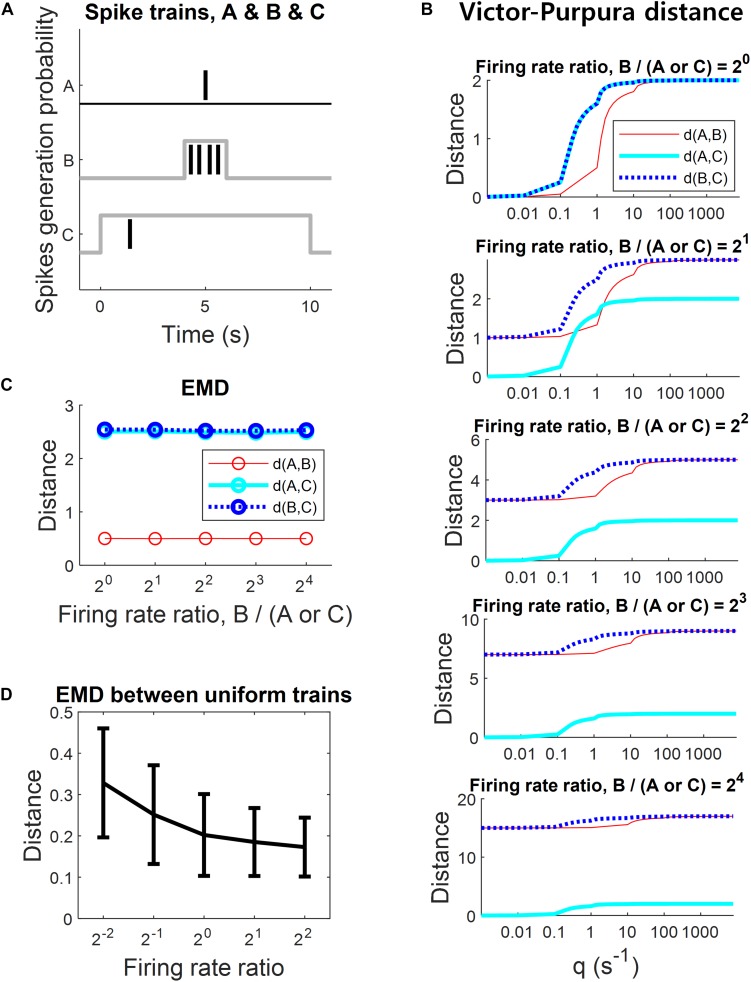
Comparison with the Victor-Purpura distance in terms of suitability for temporal coding with different firing rates. **(A)** Spike train A has only one spike with fixed timing. Spike train B has five levels of spikes with narrow range spike timing jitters. Spike train C has only on spike with a broad range spike timing jitter. The desirable expected results are that the distance between spike trains A and B is smaller than the distance between A and C and the distance between B and C. **(B)** The Victor-Purpura distance with various values of the time-scale parameter q. The Victor-Purpura distance did not show the desirable result with increases in firing rate ratio. **(C)** The EMD showed desirable results overall with increases in firing rate ratio, having a nearly constant scale. **(D)** The EMD between a uniform spike trains with different firing rate ratios. It shows that the EMD is not completely insensitive to firing rate differences.

The spike train distance in the present study is closely related to the Victor-Purpura distance. It is important to compare the properties between the Victor-Purpura distance and the EMD. Satuvuori and Kreuz (2018) already discussed the suitability of the Victor-Purpura distance to rate and temporal coding. They suggested that the Victor-Purpura distance is suitable to rate coding in general, but suitable to temporal coding only for similar firing rates, even with a wide range of time-scale parameter q. To verify whether the EMD suffered from a similar issue to the Victor-Purpura distance, we applied the analysis of Satuvuori and Kreuz (2018) to the EMD. Three spike trains were generated in the analysis. Spike train A was generated to contain one spike discharged at 5 s. Spike train B was generated according to a probabilistic model of a uniform distribution centered at 5 s with the halfwidth of 1 s. Spike train C was also generated according to a probabilistic model of a uniform distribution centered at 5 s with the halfwidth of 5 s. Spike train B had five levels of the number of spikes; 2^0^, 2^1^, 2^2^, 2^3^, and 2^4^. By comparison, spike train C had only one spike as in spike train A (see Figure 6A). From the point of view of temporal coding, it was expected that the distance between A and B was smaller than the distance between A and C and the distance between B and C, because spike trains A and B had more similar temporal information compared to C. The Victor-Purpura distance was examined for time-scale parameters in the range from 0.01 to 1000.

## Results

### Spike Timing Difference

The spike train distance measurements exhibited differences among the five spike train distances tested in this study. The Victor-Purpura distance linearly increased as the spike timing difference increased with one parameter value (q = 0.1 s), but was saturated with the other parameter values (q = 0.8, 12.8 s). Similarly, the van Rossum distance monotonically increased as the spike timing difference increased with one parameter (… = 16 s), but was saturated with another parameter (… = 1, 4 s). Both the SPIKE-distance and the RI-SPIKE-distance increased first but later decreased as the spike timing difference increased. The EMD linearly increased as the spike timing difference increased (Figure 3B).

Also, we observed that the SPIKE-distance and the RI-SPIKE-distance consider the spike trains to be more similar if a middle spike is close to the edge spikes than if the middle spike is located at an equal distance from both edge spikes. The reason is that these methods focus on the local dissimilarity between spike trains. Two spike trains are locally similar when the middle spike is close to the edge spikes since then it becomes easier to see it as part of a doublet that together is quite synchronous with the single spike in the other spike train. In contrast, if the middle spike is located at an equal distance from both edge spikes, then the distance to the nearest spike in the other train is maximized, increasing local dissimilarity. The van Rossum distance seems to evaluate a similarity of two spike trains based on synchronization of spike timings within a certain temporal range, where the temporal range was determined by the time-scale parameter …. Then, if two spikes from each spike train occurred within the temporal range, these spikes were deemed to be synchronized. The Victor-Purpura distance with the parameter q = 0.1 s (i.e., emphasizing temporal differences) and the EMD linearly increase as the difference of middle spikes is linearly increased, because these methods focus on equalizing two spike trains. Hence, for instance, if a difference in the latency of neural responses between spike trains is of interest, the Victor-Purpura distance with a small q and the EMD can provide an appropriate measure.

The characteristics of distances for small spike timing differences (for example, the middle spike is shifted by 0, 1, or 2 in Figure 3B) can provide information about temporal precision of the spike timing. The Victor-Purpura distance (q = 0.1 s) and the EMD are linearly decreasing when the spike timing difference converges to zero. This linear property allows them to have the information about temporal precision, but with no conclusive answer to whether a timing difference between spike trains is precise or not. On the other hand, the van Rossum distance, the SPIKE-distance and the RI-SPIKE-distance are rapidly decreasing when the spike timing difference is nearing zero so that they can provide precise information whether timing difference falls within some range or not.

### Temporal Similarity and Robustness to Firing Rate Change

We evaluated distance measurements between a pair of spike trains with a high or low temporal similarity when the ratio of the firing rates between the trains varied. Let D_L_ be a distance with a low temporal similarity (i.e., between A and B2) and D_H_ be a distance with a high temporal similarity (i.e., between A and B1) (see section “Temporal Similarity and Robustness to Firing Rate Change”). First, we calculated the ratios of D_L_ to D_H_ from each distance for the case when the firing rates of two spike trains were equal, and the result is summarized in Figure 4C. The Victor-Purpura distance (q = 0.1, 0.8 s), the van Rossum distance (… = 4, 16 s), and the EMD clearly resulted in a smaller distance with a high temporal similarity than with a low temporal similarity (Figure 4B). These low and high temporal similarities reflect the global difference between two spike trains in Figure 4A, not the local difference. The spike trains B1 and B2 in Figure 4A are globally different, but locally similar (near 0 s and 10 s). Since both the SPIKE-distance and the RI-SPIKE-distance focus on the local difference, these distances show less sensitivities for the discrimination between low and high temporal similarity in a global sense. In contrast, the EMD is a global measurement, showing an ability to discriminate global temporal similarity. Although the RI-SPIKE-distance is robust to firing rate changes just as the EMD is, this point indicates a key difference between the RI-SPIKE-distance and the EMD (see section “Discussion”).

Next, to assess the robustness to firing rate changes when unequal firing rates exist between the spike trains, we calculated the ratio [D_L_(i) − D_H_(i)]/| D_H_(1) − D_H_(i)|, where i denotes the firing rate ratio of spike train B1 (or B2) to that of spike train A for *i* = 1/4, 1/2, 2, 4 [e.g., D_H_(1/2) refers to distance measurements when the firing rate ratio is 1/2]. The distance results for each value of *i* are given in Figure 4D. As for the robustness to firing rate changes, the Victor-Purpura distance and the van Rossum distance increased as the ratio of the firing rates deviated from 1, which indicates that variability in the distance across the firing rate ratios was larger than the difference in distances between high and low temporal similarities, revealing that the distances were not robust to firing rate changes. This was not the case for the SPIKE-distance and the RI-SPIKE-distance, where the distances remained at similar levels across the ratios of firing rates although variability in the distance across the ratios was larger than the difference in distances between high and low temporal similarities, showing that they were also not robust to firing rate changes. On the other hand, the EMD showed that variability in the distance across the ratios was much smaller than the difference in distances between high and low temporal similarities, demonstrating its robustness to firing rate changes (Figure 4B). The SPIKE-distance, the RI-SPIKE-distance, and the EMD showed the robustness to firing rate changes relative to the Victor-Purpura distance and the van Rossum distance. It implies that those three distances are more suitable for temporal coding.

### Spike Time Synchrony and Robustness to Firing Rate Change

Spike train distances with various synchrony levels were measured using each of the five distances and their robustness to firing rate changes was tested. Every distance clearly showed a similar pattern when the ratio of firing rates was 1 such that the spike train distance increased as the degree of spike timing jitter increased (Figure 5B). To assess the robustness to firing rate changes, we quantified the effect of the firing rate ratio on the spike train. Here, let D_k_(n) be the distance when the firing rate ratio of spike train B to spike train A was n, where k denotes the halfwidth of the uniform distribution in B (see section “Spike Time Synchrony and Robustness to Firing Rate Change”). We first calculated the ratio D_0_._05_(9)/D_0_._05_(1) using each distance and obtained the results as summarized in the left figure of Figure 5D. Next, we calculated the ratio [D_0_._5_(9) − D_0_._05_(9)]/[D_0_._5_(1) – D_0_._05_(1)] using each distance and obtained results, which are listed in the right figure of Figure 5D. A comparison of these two ratios showed that when the firing rate ratio increased, the Victor-Purpura distance and the van Rossum distance increased rapidly, whereas other distances were almost unchanged. In other words, by using the Victor-Purpura distance and the van Rossum distance, variability in distance across the firing rate ratios was larger than variability in distance due to different degrees of spike timing jitter, showing that the distances were not robust to firing rate changes. The SPIKE-distance, the RI-SPIKE-distance, and the EMD revealed that variability in distance across the ratios was smaller than that among different levels of synchrony, demonstrating that they were robust to firing rate changes. Moreover, the RI-SPIKE-distance and the EMD appeared to be most robust (Figure 5C). These results indicate that the Victor-Purpura distance and the van Rossum distance are suitable to measure the dissimilarity due to both rate difference and temporal synchrony. The SPIKE-distance is also suitable to measure the dissimilarity in both rate difference and temporal synchrony although it seems to be less sensitive to rate difference than the Victor-Purpura distance and the van Rossum distance. On the other hand, the RI-SPIKE-distance and the EMD are suitable to measure temporal synchrony, insensitive to rate differences.

### Comparison With Victor and Purpura’s Distance

The simulation result for the Victor-Purpura distance in the present study was similar to that in the study by Satuvuori and Kreuz (2018). The expected result was that the distance between the spike trains A and B was smaller than those between A and C and between B and C, because the temporal coding between A and B is more similar than that between other pairs (Figure 6A). When the firing rate ratio of B to A or C was 2^0^ (i.e., the same firing rates), the Victor-Purpura distance showed the expected result for a wide range of time-scale parameters q (Figure 6B, top). It indicates that the Victor-Purpura distance is suitable for temporal coding if the firing rate ratio is 1. However, as the firing rate ratio of B to A or C increased, the Victor-Purpura distance started to show unexpected results. The distances between A and B and between B and C were increasing for every time-scale parameter q, reflecting the increased rate difference (Figure 6B). It indicates that the Victor-Purpura distance is not suitable for temporal coding if the firing rate ratio deviates from 1. The smaller value of the time-scale parameter q emphasizes the temporal coding. However, the result showed that the Victor-Purpura distance is still rate-sensitive even for a very small value of *q*. Therefore, the value of *q* apparently changes sensitivity from pure rate coding to combined rate and temporal coding, not to pure temporal coding (Satuvuori and Kreuz, 2018).

On the other hand, the EMD showed the expected results for all tested firing rate ratios. Furthermore, the distances between every pair of spike train remained nearly constant even as the firing rate ratio changed (Figure 6C). It indicates that the EMD is suitable for temporal coding even though the firing rates differ between the spike trains, showing that it does not reflect rate coding. That is, the EMD is sensitive to pure temporal coding in contrast to the Victor-Purpura distance.

Although the EMD is relatively insensitive to firing rate difference than the Victor-Purpura distance, it is uncertain whether the EMD is completely insensitive. In order to test the effect of different rate ratios on the EMD, we calculated the EMD between two Poisson spike trains that were generated uniformly over [0, 1] s with different rates. The spike trains were generated with firing rates of 1, 2, 4, 8, and 16 Hz. Then, the spike trains with 4 Hz were compared to those with other firing rates (including the identical 4 Hz) so that the firing rate ratios varied over 2^–2^, 2^–1^, 2^0^, 2^1^, and 2^2^. The resulting EMD values are provided in Figure 6D. The EMD between trains with the same temporal pattern varied across different firing rate ratios although the EMD variation was much smaller than the firing rate ratios variation.

### Application to Neural Data

**FIGURE 7:**
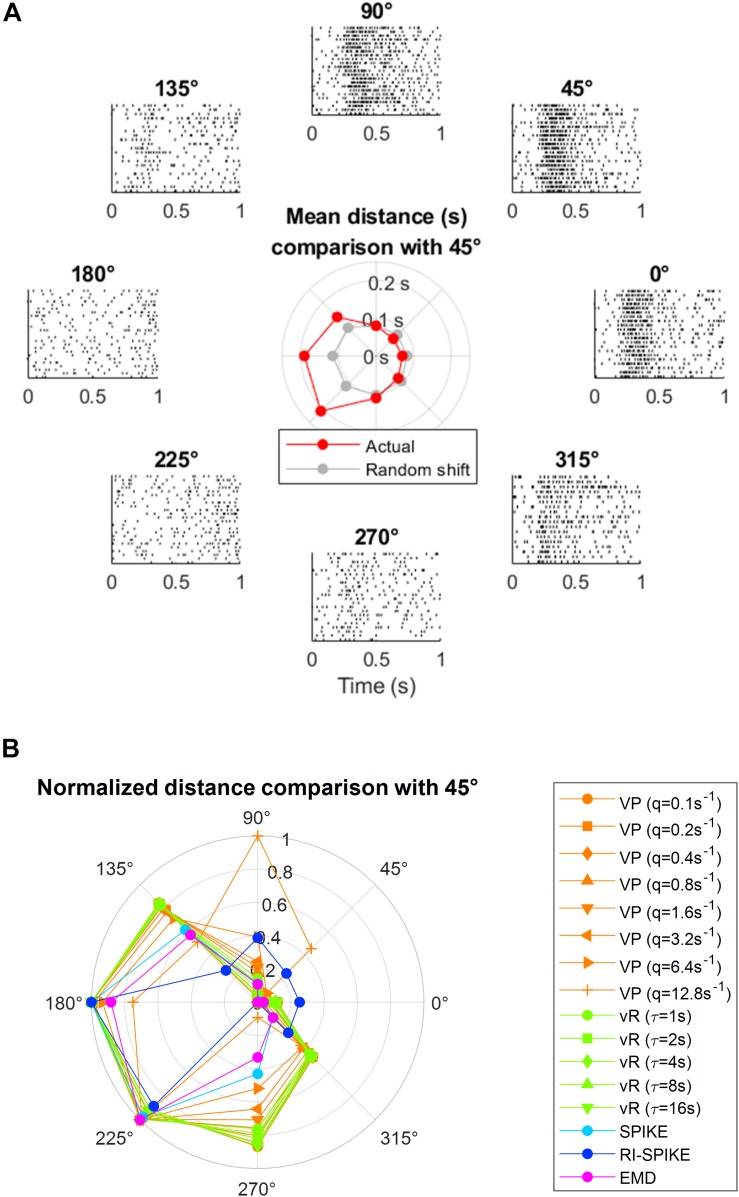
Application of the spike train distance to real neuronal data in the primary motor cortex in a non-human primate (Flint et al., 2012). During the data recordings, the subject moved its arm from the central position toward one of the eight target positions and repeated this movement multiple times for each direction. Multiple spike trains of a single neuron for each of the eight target positions are described at each peripheral location, indicated by a directional angle as 0°, 45°, …, and 315°. Each spike train is obtained for 1 s after the onset of a movement cue. The spike trains exhibit different temporal patterns for different directions. **(A)** The direction at 45° is set as the seed direction, where the firing rate is maximum. Then, the spike train distance is calculated between the seed direction and each of other seven directions. The mean spike train distance between each pair is described using red circles in the center. The spike train distance within the seed direction is also calculated for comparison (no calculation between the same spike trains). The EMD from actual data (red line of the inner graph) has a clearer difference between the base and orthogonal angles than the EMD from data of randomly shifted spike timing (gray line of the inner graph), which does not exhibit a temporal pattern, showing the EMD difference is not merely due to the firing rate differences. **(B)** Comparison between the EMD and other spike train distances for the data in panel **(A)**. Each spike distance was normalized such that D_new = (D − D_min)/(D_max − D_min) so that the distance values are filled between 0 and 1, because each spike distance has different magnitude scale. Throughout our spike distance analyses, we have set up a time-scale of spike trains for the Victor-Purpura distance and the van Rossum distance as [0, 10] s, which makes these distances applicable to both rate coding and temporal coding. To be consistent with such parameter settings of all the analyses done in the study, we also maintained the same time-scale range for the analysis of real (neuronal spike data in panel **(A)**. Since the spike trains of the real neurons we analyzed lasted for 1 s after a task onset, we extended spike trains by multiplying 10 to spike timings, changing the spike train range from [0, 1] s to [0, 10] s and used the same parameter settings as other simulation-based analyses for the Victor-Purpura and the van Rossum distances. This extension of the spike train range does not alter the SPIKE-distance, RI-SPIKE-distance and the EMD because they produce time-scale independent distance outcomes. VP, Victor-Purpura distance; vR, van Rossum distance.)

We demonstrated the measurement of a temporal similarity between real neuronal spike trains using the EMD. The neural data is publicly available from Flint et al. (2012), and can be downloaded from https://crcns.org/data-sets/movements/dream. The example of neural spike trains was obtained from the primary motor cortex of a behaving non-human primate (Flint et al., 2012). An example of various levels of temporal similarity measured by the EMD is shown in Figure 7, in which the spike trains observed under the different experimental conditions (i.e., different movement directions of the subject’s arm) showed mutually different temporal similarity with the base condition at the arm movement direction of 45° (at which the example neuron fired the most).

A neuron in the primary motor cortex (M1) modulates its firing rates with arm movement directions (Georgopoulos et al., 1982). Arm movements induce a certain temporal pattern such that a spike train of a M1 neuron contains more spikes around movement onset and less spikes before and after movement offset. Also, the firing rate of the neuron is maximal at the preferred direction (PD) of arm movement and decreases gradually when the movement direction deviates farther from the PD (Georgopoulos et al., 1982; Schwartz et al., 1988; Kalaska et al., 1989; Caminiti et al., 1990). Hence, the temporal patterns of spike trains between the PD and other directions are expected to be more dissimilar when the movement direction becomes more different from the PD. We found that the EMD could describe various levels of temporal similarity to the base condition for various directions and specifically showed that distance increased as the angle became orthogonal to the PD. In addition, the EMD on the true data (red lines in the inlet graph of Figure 7A) revealed a clearer difference between the PD and orthogonal angles than that on the surrogate data with randomized spike timings (gray lines of the inlet graph of Figure 7A). Specifically, corresponding to each true spike train, we generated a random spike train by generating spike timings from the uniform distribution while maintaining the number of spikes unchanged. So, if the difference between directions is mainly represented in the number of spikes, the difference between directions should also be maintained in the surrogate data. However, the result demonstrated that the EMD difference between spike trains of different directions was not merely due to the firing rate difference.

A spike train distance shall yield small values between spike trains obtained under similar experimental conditions and large values between spike trains obtained under different conditions. We demonstrated that the EMD satisfied such a criterion using the real neuronal spike data of a non-human primate in Figure 7. In Figure 7, the EMD showed small values when the subject moved the arm in a direction similar to the preferred direction (i.e., similar experimental condition) and large values when the subject moved the arm in a direction dissimilar to the preferred direction (i.e., dissimilar experimental condition) (see red lines in the inlet graph of Figure 7A). In particular, the EMD calculated this result based on the temporal pattern rather than on the firing rate difference.

We compared EMD and other spike train distances in terms of an ability to distinguish primary motor cortical spike trains with spiking timing information according to the arm movement directions of a non-human primate. There were eight equally divided arm movement directions in this 2D center-out arm reaching task. As each spike train distance covered a different magnitude scale, each spike train distance was normalized by D_new = (D − D_min)/(D_max − D_min) so that the distance values ranged between 0 and 1. We selected one of the eight directions as an anchor (e.g., 45°) and measured average pairwise distance using each spike distance measure between a set of spike trains corresponding to the anchor direction and each set of spike trains corresponding to other directions. We found that the EMD well represented differences between spike trains according to movement directions such that the distance is 0 at the PD, 1 at the opposite of the PD, and the intermediate values at other directions (Figure 7B).

We evaluated how the EMD could be used to discriminate the neural spiking patterns of different upper limb movement directions represented in the primary motor cortex (M1) of a non-human primate (Flint et al., 2012). The non-human primate moved the upper limb in eight different directions while spiking timings of the population of M1 neurons were recorded. There were multiple trials of this task in each direction. As the duration of movements varied across trials, we selected a 1-s epoch after the onset of a go cue. Before spike train distance computation using various methods including the EMD, we normalized the overall spike count of every spike train in order to assess each method’s ability to extract movement-related information only from spiking timing patterns. This normalization was performed based on resampling – i.e., randomly selecting a certain number of spikes from the original spike train. In this manner, every resampled spike train could have the same number of spikes for every direction while retaining the temporal pattern of the original spike trains.

**FIGURE 8:**
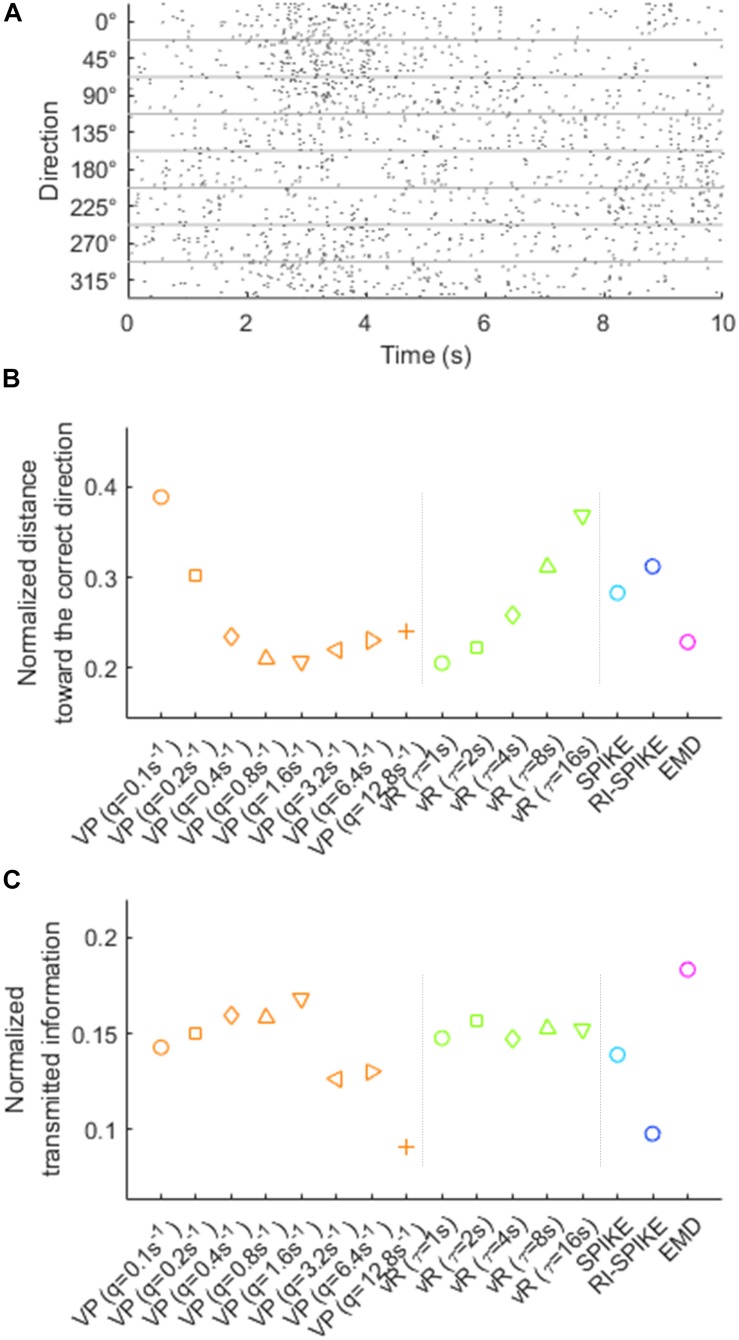
**(A)** Resampled spike trains of the neuron in Figure 7. The spikes in the resampled spike train are randomly chosen from the pool of spike timings in each direction. The spiking patterns in the original spike train is preserved while the number of spikes is controlled. The range of spike trains extended from [0, 1] s to [0, 10] s. **(B)** The directional decoding results using spiking patterns in resampled spike trains. The distance indicates the average distance for each direction. The distance for the direction is normalized by other directions. The small value of the normalized distance toward the correct direction indicates a high magnitude of discrimination of the spiking patterns for the direction from the other directions. **(C)** Decoding directional information from the spike trains in panel **(A)** is performed using the k-nearest neighbor algorithm (*k* = 3 in our analysis) and evaluated by the normalized transmitted information (see the text). Higher normalized transmitted information indicates better decoding performance. VP, Victor-Purpura distance; vR, van Rossum distance.

For resampling, we first selected 113 out of 196 M1 neurons, which fired spikes enough to produce spike trains suitable for our distance analysis (a neuron was selected if it fired ≥50 spikes within the 1-s epoch on average for each direction). For each selected neuron, we randomly chose *R* spikes from the original spike train, repeating this resampling for every spike train of every direction for that neuron. The number of spikes in a resampled spike train, *R*, was stochastically determined by generating a random number from the Poisson distribution with the mean rate of 10. The mean rate of 10 was chosen such that the largest number generated from the Poisson distribution with this mean rate was unlikely to exceed the half of 50 (i.e., 25), in order to make resampled spike trains vary over trials. This ensured that the expected number of spikes in every resampled train in every direction was identical, while allowing trial-to-trial variability. Once the resampled spike train was generated, we multiplied 10 to its spike timings to change the spike train range from [0, 1] s to [0, 10] s, in order to adjust the range adequate for pre-defined time-scale parameters of the Victor-Purpura distance and the van Rossum distance. Also, as the SPIKE-distance and the RI-SPIKE-distance calculate the distance in a range from the first spike to the last spike, we added two auxiliary spikes at 0 and 10 s (Figure 8A).

For the assessment of each spike train distance method, we randomly selected a single resampled spike train in the k-th direction and calculated distance between it and every other resampled spike trains using a given spike train distance. Those calculated distances were averaged for each direction, yielding the average distances d_i_ (i = 1,…,8) for each of the eight directions. The averaged distances were then normalized over direction such that $\bar{{\text{d}}_{\text{i}}}=\left({\text{d}}_{\text{i}}-{\text{d}}_{\text{min}}\right)/\left({\text{d}}_{\text{max}}-{\text{d}}_{\text{min}}\right)\,$ as above. The shorter normalized distance toward the correct direction, $\bar{{\text{d}}_{\text{k}}}$, represented better discrimination of the spiking patterns for the correct direction from other directions. The EMD, as well as the Victor-Purpura distance and the van Rossum distance with specific parameter settings, resulted in shorter distances than others (Figure 8B). Therefore, it demonstrated that the EMD could decode the directional information of upper limb movements in M1 neurons based on spiking timing patterns.

We applied a clustering analysis [Houghton and Victor (2010) and Victor and Purpura (1996)] to the data shown in Figure 8A in order to compare the effect of each distance metrics on decoding the information of movement directions from spike trains. For decoding such directional information based on the shortest distance to the training samples of spike trains, we used the k-nearest neighbor algorithm (Fix and Hodges, 1951). The decoding performance were measured by the normalized transmitted information proposed in the study by Houghton and Victor (2010), which ranges from 0 to 1 where a higher value indicates more accurate decoding. The result demonstrated that the EMD produced the best decoding output (with the number of neighbors, *k* = 3) (Figure 8C).

We also applied the same clustering analysis to the data shown in Figure 7A without removing firing rate differences, in order to examine the effect of directionally tuned firing rates on the spike train distances. We observed that the Victor-Purpura distance and the van Rossum distance produced larger normalized transmitted information than the SPIKE-distance, the RI-SPIKE-distance, and the EMD regardless of the setting of time-scale parameters. It demonstrates that the first two distances are more suitable for rate coding than last three distances, as also shown in sections “Temporal Similarity and Robustness to Firing Rate Change” and “Spike Time Synchrony and Robustness to Firing Rate Change.”

## Discussion

In the present study, we applied the EMD to neuroscience as a spike train distance to measure the shortest delivery path length between spike trains. In this distance, a spike train was considered as a function that assigned a non-negative value at spiking time such that the sum of all non-negative values was equal to one. For any two functions in this metric space, one function could be transformed into another function through the iteration of delivering a quantity at a point in the domain of a function to another point. Each delivery created a path whose length could be quantified by the product of the amount of the moving quantity (i.e., a fraction of a spike) and the delivery time. The sum of all delivery paths was then defined as the delivery path length. Among all possible delivery paths, the shortest path was sought, and its length was used as the spike train distance. We demonstrated that our distance sufficiently expressed temporal similarity based on the temporal profile of spiking probabilities and spiking time synchrony between a pair of spike trains, and that it was more robust to differences in absolute firing rates with a common temporal profile of spike probabilities than previous distances.

The metric EMD is induced by the metric based on the temporal events. It means that a distance between two spike trains is entirely measured from distances between spikes within those spike trains. Owing to this property, the EMD can vary linearly in response to linear changes in spike timing (Figure 3). This linear property may strengthen the reliability of the EMD for capturing spike timing differences between spike trains and allow one to easily determine how the distance would vary with spike timing variation. On the other hand, the EMD can provide information about temporal precision, but not conclusive information whether spike timing difference is in some range or not, due to this linearity.

The EMD measures a difference between two normalized spike trains, in contrast to other distances that use spike trains *per se* without normalization. This normalization allows the EMD to compare the actual temporal patterns of a pair of spike trains with negligible influences from firing rates. This property makes the EMD more robust to firing rate changes than other distances (Figures 4, 5). It is expected that temporal coding research may take advantage of this property.

Of course, not every spike train distance should be robust against firing rate change. If a distance between spike trains with similar firing rates is smaller than between spike trains with different firing rates, it is suitable for representing rate coding. Yet, if certain cases require information merely from temporal coding, the robustness against firing rate change would be necessary for distance measures. Since the EMD does not reflect rate coding as discussed above, the spike counts would be supplementary to the EMD.

The EMD as a spike train distance is based only on spike timing differences, which allows the EMD to be adequate for temporal coding. The EMD measures the minimum length of spike timing shifts to make two spike trains identical. To calculate the length, the amount of spikes in two spike trains should be the same and the normalization step making the amount of spikes in each train equal to one is necessary. The normalization step plays a crucial role in the robustness to firing rate changes. Thus, existing methods other than the EMD can be applied to normalized spike trains, which would preserve the robustness to firing rate changes. However, other methods may not be as adequate as the EMD for temporal coding. For example, the Jensen–Shannon divergence, which measures dissimilarity between two probability distributions, can be applied to the normalized spike trains because the normalized spike trains can be considered as a probability distribution. But, since it is not concerned with spike timing differences, it may not provide measurements useful for temporal coding. Also, the Victor-Purpura distance can be applied to the normalized spike trains. However, since the Victor-Purpura distance has the adding/deleting spikes option, it cannot guarantee that the dissimilarity is calculated based only on spike timing differences. Hence, we suggest that the EMD can be advantageous over other methods to provide spike train distance based solely on timing differences between a given pair of spikes and therefore useful for temporal coding schemes.

Precise spike timing is a key element in temporal coding (Butts et al., 2007; Gollisch and Meister, 2008; Johansson and Flanagan, 2009). There are largely two different approaches to measure how much the spike timings of a pair of spike trains match with each other. One way is to measure a global difference between the trains of spike timings, and the other way is to measure local matches between the trains of spike timings. For example, in the global measurement, a spike train (2,3,4,5) can be matched with a spike train (1,2,3,4) by shifting all spike timings by +1, and a spike train (1,2,3,5) can be matched with the spike train (1,2,3,4) by shifting its last spike timing by −1. Therefore, the distance between (1,2,3,4) and (2,3,4,5) is larger than the distance between (1,2,3,4) and (1,2,3,5). The Victor-Purpura distance with a short time-scale parameter and the EMD measure spike distances in this way: 0.4 vs. 0.1 in the Victor-Purpura distance with q = 0.1, and 1 vs. 1/4 in the EMD. In the local measurement, the spike train (1,2,3,4) and the spike train (2,3,4,5) are locally matched at three different timings ({2, 3, 4}), and the spike train (1,2,3,4) and the spike train (1,2,3,5) are also locally matched at three different timings ({1, 2, 3}). As such, the distance between (1,2,3,4) and (2,3,4,5) is the same as the distance between (1,2,3,4) and (1,2,3,5). The van Rossum distance, the SPIKE-distance, and the RI-SPIKE-distance measure spike distances in this way. Hence, we can select the global spike train distance measurement if we intend to measure how similar the distributions of spike timings in two spike trains are, or the local spike train distance measurement if we intend to focus on local spike timing matching. In this study, we propose a new method for the global spike train distance measurement.

Although the EMD is less affected by firing rate changes compared to other distances, it is not completely invariant to firing rate changes. For instance, in our simulations, when two spike trains were generated from uniform distributions in an interval from 0 to 1 s with a firing rate of 1 Hz, the EMD between two spike trains was 0.33 ± 0.24 s. However, when two spike trains were generated in the same interval with a firing rate of 10 Hz, the distance decreased to 0.14 ± 0.06 s. Therefore, there was a tendency for the EMD to decrease as the firing rate increased. Also, these results were confirmed with Poisson spike trains in Figure 6D. The results showed that the EMD values varied with different firing rate ratios although the variation was very smaller than firing rate ratios. Therefore, meticulous care is required when using the EMD for temporal coding research without considering rate coding completely. However, in cases where two spike trains exhibit certain temporal patterns and those two temporal patterns are different, the EMD would quantify dissimilarity well between two temporal patterns even if they have fairly different firing rates.

The present study mainly addressed the sensitivities of spike train distances to rate and temporal coding. However, in addition to sensitivities, each distance offers a unique feature. The SPIKE-distance and the RI-SPIKE-distance have fine time resolutions and thus can measure differences in local spike patterns.

The EMD also has a definite advantage such that it can be extended to stochastic spike trains as follows. Many noise sources perturb the generation of spikes, inducing a variability of spiking events (Faisal et al., 2008). Due to this variability, we can consider a spike train as a stochastic process. For examples, the peristimulus time histogram based on the average of trials or the probabilistic reconstruction of a spike train (Kass and Ventura, 2001) takes stochastic spike trains into account. In this sense, a spike train distance that can deal with continuous data is needed to compare two stochastic spike trains. Moreover, such a spike train distance should be based on distance metrics defined with deterministic spike trains, as a stochastic spike train can be viewed as a natural variant of a deterministic spike train (Haslinger et al., 2009). The proposed distance, EMD, bases itself in a metric space for deterministic spike trains and can also be applied to stochastic spike trains in the form of normalized continuous data.

## Data Availability Statement

The datasets generated for this study can be found in the [CRCNS (Flint et al., 2012)] (https://crcns.org/data-sets/movements/dream).

## Author Contributions

Both authors designed the study and wrote the manuscript. DS performed simulation and analyzed the data.

## Conflict of Interest

The authors declare that the research was conducted in the absence of any commercial or financial relationships that could be construed as a potential conflict of interest.
